# Circadian humidity fluctuation induced capillary flow for sustainable mobile energy

**DOI:** 10.1038/s41467-022-28998-y

**Published:** 2022-03-11

**Authors:** Jiayue Tang, Yuanyuan Zhao, Mi Wang, Dianyu Wang, Xuan Yang, Ruiran Hao, Mingzhan Wang, Yanlei Wang, Hongyan He, John H. Xin, Shuang Zheng

**Affiliations:** 1 Department of Chemistry, Hong Kong University of Science and Technology, Hong Kong, China; 2 Institute of Textiles & Clothing, Hong Kong Polytechnic University, Hong Kong, China; 3 Beijing Key Laboratory of Ionic Liquids Clean Process, Institute of Process Engineering, Chinese Academy of Sciences, 100190 Beijing, China; 4 University of Chinese Academy of Sciences, 100049 Beijing, China; 5 Beihang University, 100191 Beijing, China; 6 School of environmental engineering, Yellow River Conservancy Technical Institute, 475004 Kaifeng, China; 7 Pritzker School of Molecular Engineering, University of Chicago, Chicago, IL 60637 USA; 8 Department of Biomedical Sciences, City University of Hong Kong, Hong Kong, China

**Keywords:** Nanoscale materials, Fluid dynamics

## Abstract

Circadian humidity fluctuation is an important factor that affects human life all over the world. Here we show that spherical cap-shaped ionic liquid drops sitting on nanowire array are able to continuously output electricity when exposed to outdoor air, which we attribute to the daily humidity fluctuation induced directional capillary flow. Specifically, ionic liquid drops could absorb/desorb water around the liquid/vapor interface and swell/shrink depending on air humidity fluctuation. While pinning of the drop by nanowire array suppresses advancing/receding of triple-phase contact line. To maintain the surface tension-regulated spherical cap profile, inward/outward flow arises for removing excess fluid from the edge or filling the perimeter with fluid from center. This moisture absorption/desorption-caused capillary flow is confirmed by in-situ microscope imaging. We conduct further research to reveal how environmental humidity affects flow rate and power generation performance. To further illustrate feasibility of our strategy, we combine the generators to light up a red diode and LCD screen. All these results present the great potential of tiny humidity fluctuation as an easily accessible anytime-and-anywhere small-scale green energy resource.

## Abstract (web summary)

Droplet generators convert mechanical movements of droplets into small-scale electricity. Here, Tang *et al*. report a humidity-driven power generator by utilizing daily humidity fluctuation in atmosphere enabling continuous generation of electricity upon moisture absorption and desorption cycles.

## Introduction

In recent years, several drop-based generators that convert liquid moving^1–5^, such as droplet spreading, falling, bouncing, or dragging, into small-scale electricity have been introduced with different advantages. In one case, organic or water drop moving on graphene could output a voltage of a few millivolts due to the so-called drawing potential effect^1^, which depends on the motion velocity. In another case, a strategy containing impinging water drops that spread over a polytetrafluoroethylene film was designed to harvest hydraulic power with low water ^2^. In addition, a liquid–liquid triboelectric nanogenerator on the basis of drop falling across liquid membrane is also fabricated with ultra-high charge collection efficiency^3^. These works use dynamic drops, such as rains, to harvest instantaneous electric power and indicate promising prospects of drop-based generators.

**Fig. 1:**
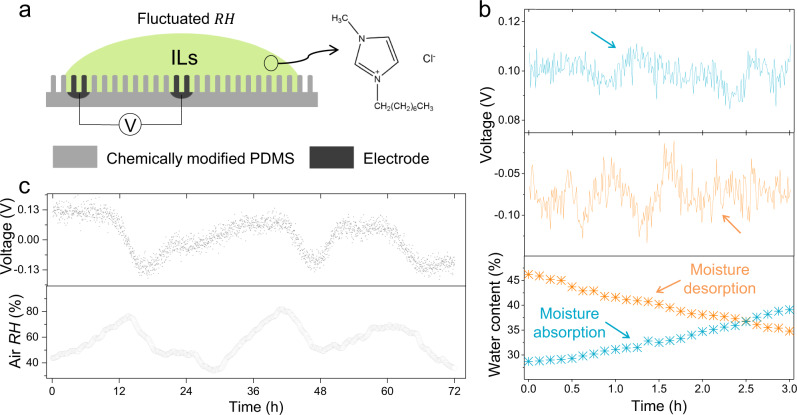
Designing details and outdoor tests of the drop-based power generator. **a** Schematic illustration of the device with IL drop pinned on PDMS nanowire array and molecular structure of the selected IL. **b** *V*_OC_ and IL drop WC curves indicating strong relation between moisture adsorption/desorption and power generation. **c** Continuous recording of *V*_OC_ from above device in outdoor tests for 3 days. The surrounding RH is also provided.

Daily humidity fluctuation is the cyclical variations of air humidity, a universal natural phenomenon ongoing all over the world^6–8^. In this work, we demonstrate that small humidity fluctuation in the air is able to trigger capillary flow within static ionic liquid (IL) drops and continuously outputs electric power, a different power generation mechanism from previous works^1–3,8,9^. When a fluid is placed on solid surfaces, the fluid wets the solid with an equilibrium contact angle (CA) *θ*_C_, defined by the Young’s equation^10–14^. Our design is such that a well-shaped fluid drop containing IL (1-Octyl-3-methylimidazolium chloride), selected due to negligible evaporation losses alongside their large moisture absorption/desorption capability when exposed to air^15^, is pinned on poly(dimethylsiloxane) (PDMS) nanowire array with certain CA (Fig. 1a and Supplementary Fig. 1). The nanowire array, with the diameter of ~90 nm and length of ~2 μm, was chemically modified to enhance surface charge. Electrode (diameter of ~800 μm) was fixed respectively to both center and edge at the bottom of the drop to collect the power. Our concept for integrating IL drop with humidity fluctuation to generate electricity is based on the following hypothesis: (1) moisture absorption/desorption within IL drop would cause directional flow along solid/liquid interfaces, (2) such flow would induce a potential difference due to ion re-distribution.

## Results

### Drop-based generators under outdoor conditions

First, to verify feasibility of our designing strategy, we use digital multimeter to collect open-circuit voltage *V*_OC_ of this device after we expose this device to outdoor air without direct sunlight for two days to ensure adequate interaction between IL and air RH before continuous test in natural environment. When the water content (WC) is below saturated water content (WC_S_), the drop tends to uptake moisture from the surroundings. As shown in Fig. 1b, WC increases from 28.7% to 39.1% with the peak *V*_OC_ reaching ~110 mV, which is in striking contrast to the *V*_OC_ in the opposite direction (peak value of −128 mV) alongside with decrease of WC. This observation indicates strong correlation between moisture absorption/desorption and power generation. To provide further support for our proposal that air RH fluctuation could induce continuous potential difference within IL drop, we record *V*_OC_ across above device for 3 days. Figure 1c shows that the drop could provide power continuously and the voltage ranges from approximately −0.15 to 0.14 V when RH fluctuates between 35 and 81%. This sustainable power supply is attributed to continuous WC_S_ variation created by the fluctuating RH that ensures the bulk IL is either unsaturated or oversaturated. Our work contrasts with previously reported humidity related generators whose power generation only lasts for several seconds or hours due to the rapidly saturated solid film^9,16–19^. All these results confirm that air humidity fluctuation could become reliable energy sources when combined with our drop-based generators.

### Drop-based generators under indoor conditions

**Fig. 2:**
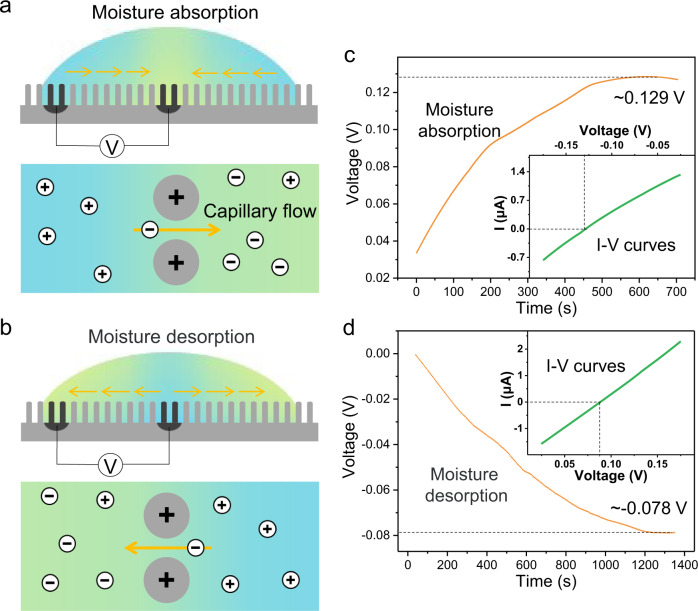
Moisture absorption/desorption induced power generation under constant temperature and humidity. **a**, **b** Schematics showing the proposed process of moisture absorption/desorption caused directional inward/outward flow and the flow induced potential difference. **c**, **d** *V*_OC_ of low-/high-water-content IL drop exposed to constant air RH of ~40% at 25 °C. Inset was the I–V curves.

To exclude other interference factors, such as wind or temperature fluctuation, we conduct windless indoor experimental research under constant temperature (~25 °C) and RH (~40%), which means a fixed WC_S_ for 1-Octyl-3-methylimidazolium chloride. When beginning to investigate how IL drop interacts with air moisture, there are three possible situations. In the first case, the bulk IL WC is lower than WC_S_, such as a dry IL drop placed on the nanowire array with the initial WC close to 0% (Supplementary Fig. 2a). The drop tends to absorb atmosphere water and expands continuously, while pinning by nanowire array prevents advancing of contact line. To keep the spherical cap regulated by surface tension, we predict that there is a flow from edge to center to remove excess fluid around the perimeter (Fig. 2a and Supplementary Fig. 2c), which gives rise to a net charge transport along electrical double layer and a relatively stable potential difference at the solid/liquid interfaces. The streaming potential is monitored and it approaches ~129 mV after 600 s (Fig. 2c and Supplementary Fig. 2e). The opposite of above situation is illustrated in Supplementary Fig. 2b. When WC is higher than WC_S_ (Fig. 2b and Supplementary Fig. 2d), outward flow within the drop is supposed to generate an opposite voltage and current (Fig. 2d and Supplementary Fig. 2f), which is confirmed by continuous electrical measurement. We note that dry and wet drops show short-circuit current (*I*_sc_) of around 2 and −1.4 μA respectively (Supplementary Fig. 2g), which is much higher than previous humidity-related generators^9^. We also consider the case when WC is equal to WC_S_. Both streaming potential and current are close to 0 (Supplementary Figs. 2h, i), which is consistent with the I–V curves. These experiments indicate that the streaming potential is triggered by moisture absorption/desorption.

### Moisture absorption/desorption induced capillary flow

**Fig. 3:**
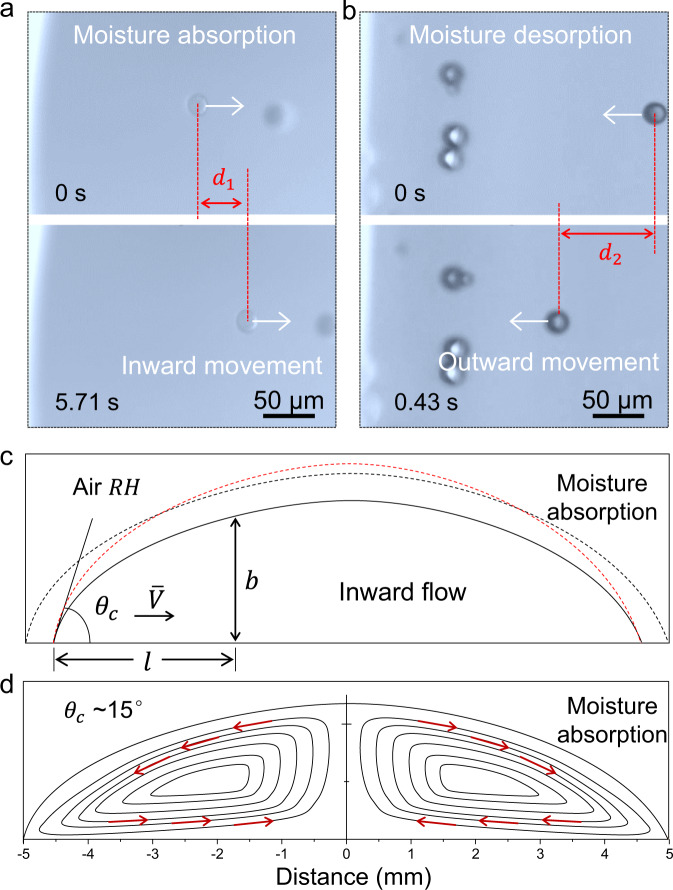
Optical observation and theoretical calculation of the directional flow. **a**, **b** In situ microscope imaged polystyrene microspheres (diameter, ~18 μm) added to dry/wet IL drop exposed to air (RH, ~40%) to confirm the inward flow and outward flow. **c** Schematic illustration and calculation of how moisture absorption could cause inward flow along the bottom of IL drops. **d** Schematic showing the moisture absorption induced complete flow within the outline of the drop recorded by microscope imaging (Supplementary Fig. 3a, c).

Next, to experimentally validate our first hypothesis that moisture absorption/desorption is able to induce flow within the IL drop, we conducted in-situ microscope imaging with microspheres included. As shown in Fig. 3a, b (Supplementary Movies 1 and 2), inward flow of 18 μm microspheres from the edge to center (*d*_1_) and outward flow (*d*_2_) at inverse direction were captured as dry/wet IL drop were exposed to air humidity (RH, ~40%), respectively. Taking the first case as an example, unsaturated dry IL drop (black solid line) tends to hold more water than they have done (Fig. 3c), causing drop expansion that may happen in two ways^20,21^. One is symmetrical expansion with unlimited contact line advancing (black dotted line), which is not allowed in our experiment. The other one is pinning of contact line enabled asymmetrical expansion (red dotted line). In this case, absorption flux *J(l)* causes increase of *b(l)* at the point *l*. And the variation ∆*b(l)* at the edge is 0 as constrained by the contact line, much smaller than that at the center. To achieve this, there has to be a flow that transports the liquid away from the periphery.

The flow velocity can be evaluated with the flux *J(l)*. Interaction of IL drop with surrounding atmosphere causes rapid decrease of water concentration in adjacent air (*μ*_a_), which, at the earlies stage, is equal to the saturated humidity of initial dry IL (*μ*_S_). While at the infinity, the air water concentration is the ambient humidity. According to the normal diffusion law, the flux is defined by:

1 $$J(l)=-{{{{{\rm{D}}}}}}\nabla {{{{{\rm{\mu }}}}}},$$

where D is the diffusivity. To remove the excess fluid near the perimeter, there need to be a flow along the bottom of the drop with the velocity^20^:

2 $$\bar{V}\propto J(l)$$

In addition to the bottom flow, we also found the inverse flow along the top layer of the drop (Fig. 3d and Supplementary Fig. 3 and Supplementary Movies 3 and 4), which we attribute to the Gibbs–Marangoni effect^22–24^.

In contrary to moisture absorption induced bottom inward flow, moisture desorption reverses the flow direction with similar reasons. In detail, moisture desorption at the drop edges is faster because of the larger relative surface area compared with that at the center. To maintain the surface tension-regulated spherical cap profile, fluid loss from the edge must be replenished by fluid from the interior due to the contact line pinning caused by nanowire array. Therefore, outward flow arises to transport excess fluid from the center to the perimeter Supplementary Fig. 3b, d.

**Fig. 4:**
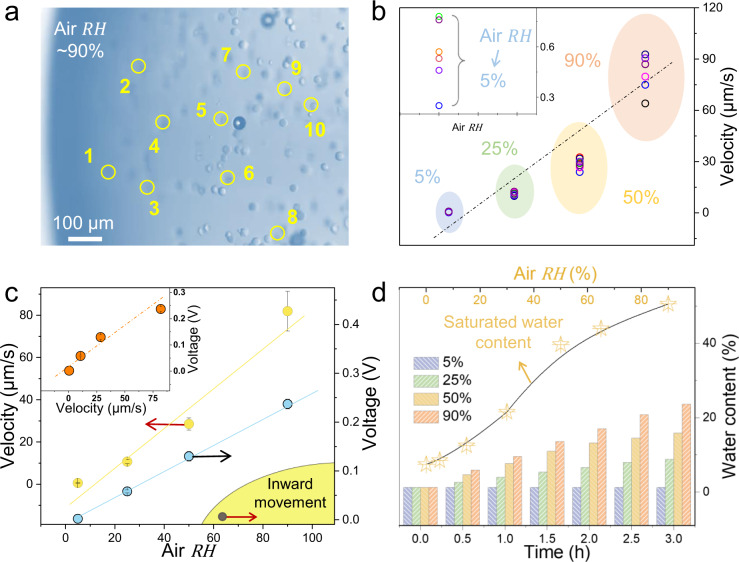
Dependence of flow velocity and output voltage on air humidity. **a** Snapshot of polystyrene microspheres movement of dry IL under air humidity of 90%. **b** Statistics of velocity under RH of 5–90%. **c** Averaged velocity and *V*_OC_ versus RH. Inset is dependence of *V*_OC_ on inward movement velocity (0–80 μm/s). **d** WC of dry IL drop versus time and WC_S_ under different RH (25 °C).

Further, to validate our second hypothesis and provide further insight into the relationship between moisture fluctuation and directional flow, we expose above dry drop to air with RH ranging from 5 to 90% and conduct statistical analysis of movement velocity of the microsphere near the edge (Fig. 4a). Quantification of depth-averaged velocity revealed a tendency versus air RH at the early stage. For example, under the RH of 5%, movement velocity fluctuated in a range of 0.2–0.8 μm/s, and this value is improved to 65–100 μm/s under the *RH* of ~90% (Fig. 4b and Supplementary Fig. 4). This observation of moisture-dependent microspheres transport is consistent with above-described Eqs. (1) and (2) as increase in surrounding water concentration *μ*_a_ results in larger gradient *∇μ* and finally accelerates the movement $\bar{V}$ (Fig. 4c). We also detected the initial voltage under different RH and at the early stage, there was a positive correlation between flow rate and *V*_OC_, in agreement with previous observations^25,26^. The water absorption speed was quantified by weighing the IL versus time. As shown in Fig. 4d, moisture content of IL drop under RH of ~90% was much higher than other cases. In addition to a constant humidity, we also exposed our drop to dynamically changing humidity using moisture generator (Supplementary Movies 5 and 6). It showed that improving the surrounding humidity would obviously accelerate the flow with higher output voltage (Supplementary Fig. 5).

### Mechanism of capillary flow induced power generation

**Fig. 5:**
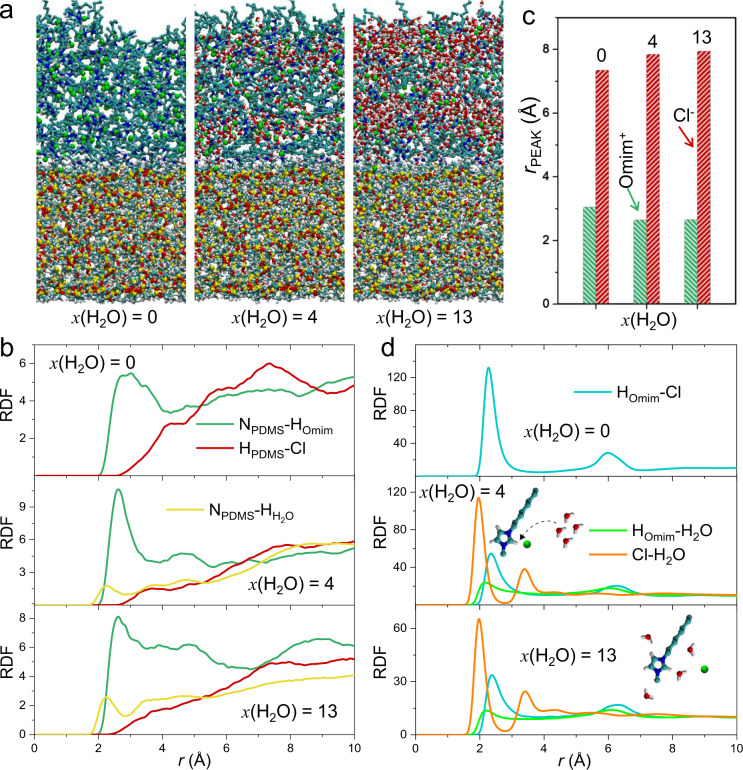
Moisture influenced N_PDMS_-Omim^+^ and Omim^+^-Cl^−^ interactions. **a** The interfacial model of N_PDMS_ and IL-water mixtures in the MD simulations. **b** The RDF for various atom pair: N_PDMS_–Omim^+^ (dark green), N_PDMS_–Cl^−^ (red), and N_PDMS_–H_2_O (yellow). **c** The position of the first peak of RDF between ILs and PDMS for various water content. **d** The RDF of the atom pair: Omim^+^–Cl^−^ (blue), Omim^+^–H_2_O (bright green), and Cl^−^–H_2_O (orange).

We subsequently conducted molecular dynamics (MD) simulations to demonstrate moisture-mediated surface grafted N_PDMS_-IL interactions and moisture-destructed cation-anion interactions within IL successively (Fig. 5a). Radial distribution function (RDF)^27,28^ represents how density of certain atom varies as a function of distance from a reference atom, typically investigated for assessing the probability of target atom to interact with the reference atom. Firstly, we determine RDF in Fig. 5b for the N_PDMS_–H_Omim_ and N_PDMS_-Cl^−^ pair, where N_PDMS_ and H_Omim_ represent the N atom and H atom grafted onto nanowire array and cation Omim^+^, respectively. As water content *x*(H_2_O) varies from 0 to 13 (*x*(H_2_O) = mol(H_2_O)/mol(IL)), the RDF peak position of N_PDMS_–H_Omim_ shifts from 3.04 to 2.65 Å while that of N_PDMS_-Cl^−^ varies from 7.35 to 7.95 Å (Fig. 5c), suggesting the enhanced interaction between PDMS nanowires and Omim^+^ and the weakened interaction between PDMS nanowires and Cl^−^.

Meanwhile, we summarize the RDF for cation–anion ion pair in Fig. 5d, where the peak position of Omim^+^–Cl^−^ RDF increases and that of Cl^−^–H_2_O decreases as *x*(H_2_O) rises. The absorbed water molecule shows high affinity to Cl^−^ and destroys the hydrogen bond between cation and anion in ILs, which agrees well with the experimental results (Supplementary Fig. 6). Overall, moisture-enhanced Omim^+^ affinity to chemically modified PDMS nanowires integrated with moisture-decreased Omim^+^/Cl^−^ bonding lays the foundation for flow-triggered cation–anion redistribution and potential difference over PDMS surfaces^29,30^.

**Fig. 6:**
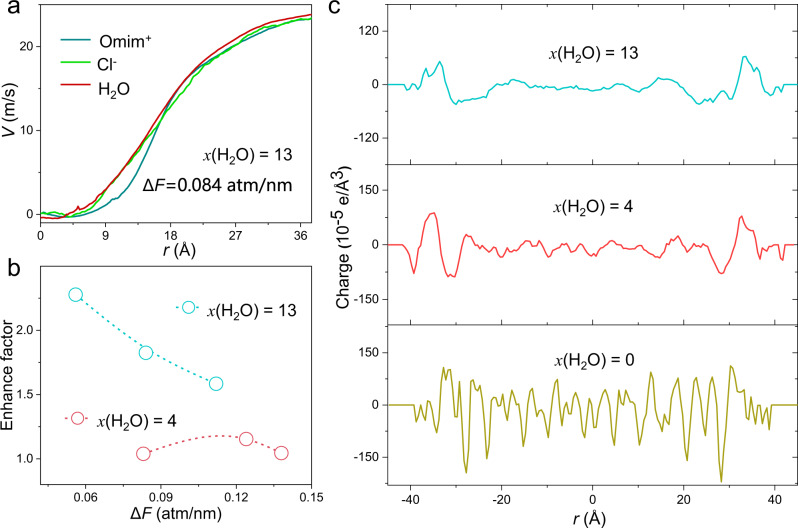
Flow induced potential difference across PDMS nanowire array. **a** The velocity distribution of Omim^+^, Cl^−^, and H_2_O confined within PDMS walls under pressure gradient of 0.084 atm/nm. **b** The enhance factor for ILs with *x*(H_2_O) = 4 and 13 as a function of the pressure gradient. **c** The charge distribution as a function of distance between the two PDMS layers when *x*(H_2_O) is 0 (bottom), 4 (middle), and 13 (top).

When above moisture-separated loose IL ion pair flows over modified PDMS nanowire array driven by surface tension gradient, nonequilibrium MD simulation reveals that asymmetric movement of Omim^+^ and Cl^−^ causes charge accumulation and electrical potential difference (Supplementary Figs. 7 and 8), in strong contrast to intimately paired IL ions without solvent moisture. Specifically, for applied pressure gradient Δ*F* = 0.084 atm/nm (Fig. 6a), the velocity of Cl^-^ is obviously higher than Omim^+^ within 1.5 nm distance from the wall, obviously different from the behaviors of bulk IL. To compare the variation of cation and anion velocity caused by different flows, the enhance factor (*ε* = v_Cl_-_,wall_/*v*_Omim+,wall_) dependence of pressure gradient was illustrated in Fig. 6b. Surprisingly, we find the enhance factor for *x*(H_2_O) = 13 is far larger than that for *x*(H_2_O) = 4, especially when the pressure gradient is small. The movement difference between cation and anion near PDMS wall indicates the possibility of flow-triggered charge accumulation.

In addition to moisture-mediated intermolecular interactions in Fig. 5, the nano-confined space within PDMS nanowire array also restricts the movement of Omim^+^, which aggregates dynamically to form clusters under hydrophobic interactions. We find from Supplementary Fig. 9, larger ionic clusters (containing ~170 atoms) form in bulk IL with both *x*(H_2_O) = 4 and *x*(H_2_O) = 13. Overall, slowed movement of moisture-clustered Omim^+^ within confined PDMS nanowire array gaps integrated with moisture-enhanced affinity of Omim^+^ onto nanowire walls enables Omim^+^/Cl^−^ separation upon flows across PDMS/IL interfaces. MD simulation shows the interfacial charge stratification when *x*(H_2_O) = 4 and 13 (Fig. 6c), in contrary with *x*(H_2_O) = 0 that the charge distribution oscillates wildly due to the not further distinguished migration of the intimate ion pairs. All these results indicate the critical role of water for our power generation mechanism.

In natural environments, wind is another important factor that may interact tightly with the IL drop-based device. As it turns out, the streaming potential in Fig. 2c, d is further improved to 232 mV and 168 mV for moisture absorption and desorption respectively when exposed to wind (~2.5 km/h) possibly because of the air flow that dynamically removes the air contacting the drop surfaces and thus accelerates the moisture absorption/desorption process. To provide more evidence for this hypothesis, we quantify the water absorption/desorption rate, respectively. The results in Supplementary Figs. 10 and 11 demonstrate that, under the wind speed of ~2.5 km/h, moisture absorption and desorption are improved from 3.02 and 2.95 mg/h to 4.57 and 4.72 mg/h. Above results confirmed that performance of our generator associates strongly with surrounding atmosphere.

### Demonstration of application

Finally, we illustrate utility of humidity fluctuation as an energy resource by integrating the drop-based device with commercial capacitors. First, we show that a single drop could quickly (within 1 min) charge capacitors from 1 to 1000 μF (Supplementary Fig. 12a). Second, a well-designed circuit (Supplementary Fig. 12b, c) connecting 16 capacitors (22 μF) in series was prepared to store the energy. This system finally output a voltage of up to ~2.18 V and lighted up a red LED with the working potential of ~1.8 V. In addition, we also connect the drop array for powering LCD screen with the working voltage of 1.5 V (Supplementary Movie 7). Overall, these results lay the foundation of humidity fluctuation as a new kind of low-cost green energy resource without strict demand for particular geography or climate.

## Discussion

Overall, the experimental and theoretical results presented above support our conclusion that circadian humidity fluctuation can be regarded as another clean energy resources using the two-step processes involving the humidity fluctuation induced directional capillary flow and the flow induced power generation. We hope this finding would expand the potential utility of rarely studied humidity fluctuation, differing from other natural environmental conditions, such as the widely used solar irradiance and wind energy. We wish to emphasize that humidity fluctuation is easily accessible anywhere and anytime, in contrary to solar and wind energy that relies on sunlight and strong air flows. Further efforts will seek to improve power generation efficiency for large-scale energy applications through material and interfacial designs^31,32^. In addition, the suitability for small-scale energy powering, such as flexible electronics, will also be evaluated.

## Methods

### Atomic model and computational details

The large-scale atomic/molecular massively parallel simulator (LAMMPS) is chosen to perform the MD simulations to study the characteristic of the structure and flow behavior of ILs. The water molar ratio in ILs is 0, 4, and 13, which could enough cover the day and night humidity changes. The PDMS basement is aminated and the area density of functional group is 1.25 #/nm^2^. Nine models were constructed, including SY_0/4/13_, SY_m0/4/13_, SYD_m0/4/13_, where 0/4/13 is the water content in IL, *m* represents modified PDMS basement, *D* represents two PDMS basement. The sizes of three dimensions of the PDMS basement are close to 4.5 × 4.5 × 3.6 nm^3^. The length of nanochannel in SYD_m0/4/13_ approximates to 7.8 nm.

Periodic boundary conditions (PBC) are applied in *x* and *y* direction, and open boundary is used in *z* direction. Data of 5 ns is generated for analysis after the system equilibrium in NVT ensemble. The Nose-Hoover thermostat keeps the temperature at 300 K. Time step is 2 fs. A coarse-grained model (the −CH_2_ and −CH_3_ groups in [Omim]^+^ are simplified as the united atoms (UA)) is applied to represent the cation Omim^+^, because the high-frequency vibrations contribution of hydrogen atoms is tiny^33–36^. The nonpolarizable all-atom optimized potentials for the liquid simulation (OPLS-AA) force field, SPC/E model, and CVFF force field are adopted for IL, H_2_O, and modified PDMS, respectively, which are all widespread used^37–39^. The charge of atoms in IL is scaled by a factor of 0.8 considering the effect of polarization between cations and anions^40^. The interatomic interaction parameters are calculated by the arithmetic mixing rules. Van der Waals interactions are modeled by 12-6 Lennard–Jones potential, and the cutoff is 1.2 nm. Long-range coulombic interactions are computed by particle-particle particle-mesh (PPPM) algorithm.

### Simulation details of flow

Analysis of the radial distribution function (RDF) and cluster is based on VMD and OVITO open-source software^41,42^, respectively. Average applied pressure gradient is shown in the following equation

3 $$\Delta {\mathbf F}=\frac{{{N}}\,\times\,{{f}_{0}}}{{{S}}\,\times\,{{L}}},$$

where *N* is the total atomic number of water molecules in the system, *f*_0_ is the force added to every atom of water, and *S* and *L* are the cross-sectional area and length of the nanochannel respectively. Due to the solvation and viscosity of water-IL mixture, the cation and anion will also start to move along the water. Then the velocity distribution of cation and anion in system SYD_m4&13_ can be further obtained. Considering the effect of PDMS and formation of cluster, the velocity of cation and anion within the interface possess an obvious difference. Hence, the enhance factor is proposed, that is the ratio of average anion velocity (*v*_an_) to cation velocity (*v*_ca_) within 1.5 nm distance from the wall.

### Fabrication of the nanowire array

We prepared PDMS prepolymer mixture (Dow Corning) by mixing 5 g prepolymer (Sylgard 184) with 0.5 g curing agent, followed by hand stirring for 5 min and degassing in vacuum for 30 min successively. Fabrication of the device was conducted according to Supplementary Fig. 1a. Specifically, two drops containing 0.2 μL conductive adhesive (SPI) alongside with the Ag wires were deposited onto AAO (2 cm × 2 cm, pore diameter of 90 nm, depth of 2 μm, TopMembranes Inc.) with the distance of 0.4 cm. The AAO was rinsed before using with water (18 MΩ cm, Merck Millipore), acetone (99%) and ethanol (95%) for 6 min, respectively. After curing under 80 °C (MS7-H550-Pro) for 2 h, the solid Ag was roughened by abrasive paper (P2000, Suisun Company) to enhance adhesion between Ag and PDMS substrate, which was prepared by casting and leveling of 0.2 mL prepolymer mixture before curing under 80 °C for 3 h. Finally, we removed the AAO template using 1 M aqueous NaOH at 60 °C and got the PDMS nanowire array with carefully embedded solid Ag. The device was cleaned with water and ethanol before chemical modification. Ag/AgCl electrode was prepared on the basis of this device.

### Fabrication of the drop-based generator

To enrich surface functional group, we first activated the PDMS substrate nanowire array with oxygen plasma at 100 W for 20 s and then immersed the as-prepared PDMS into (3-aminopropyl) trimethoxysilane (97%, Aladdin) solutions (1 wt%) for 20 min to allow condensation of silanol. The whole device was repeatedly rinsed with ethanol to remove unreacted chemicals and dried in air for 25 min. To enable stable pinning of IL drop on above PDMS nanowire array, we deposited 75 μL (1-Octyl-3-methylimidazolium chloride) to the center electrode and exposed the device to air with saturated humidity within a polymethyl methacrylate container for two days to ensure full water absorption. The drop diameter approaches ~1 cm and the IL could be dried in vacuum oven overnight.

### Characterization of the generator

SEM imaging was conducted with Nova NanoSEM 450 at an acceleration voltage of 5.0 kV and probe current of 15 μA. EDS characterization of chemical elements distribution was finished at the acceleration voltage of 10.0 kV. X-ray photoelectron spectroscopy (PHI5802) measurements were performed with 300 W Al Kα radiation. We investigated the capillary flow by adding polystyrene microspheres (diameter of ~18 μm, Sigma-Aldrich) into 1-Octyl-3-methylimidazolium chloride followed by hand stirring for 5 min and ultrasonic dispersion (Kun Shan Ultrasonic Instruments, KQ-100E) for 10 min. Optical observation of the polystyrene microsphere movement was recorded with a computer-connected Nikon microscope (Eclipse Ni-U). Water content and weight of the IL drop were characterized using analytical balance (ME54T, Mettler-Toledo International Inc.) with real-time recording.

### Power generation measurement under different environments

The open-circuit voltage and short-circuit current were recorded with a digital multimeter (DMM6500, Keithley Instruments). We controlled surrounding environments in different ways according to experimental requirements. For outdoor tests (Fig. 1), the device was exposed to outdoor environment without direct sunlight for two days to exclude photovoltaic effect. Then we expose the device to unadjusted outdoor physical environments for evaluating the powering performance under ever-changing conditions. In brief electric or optical measurements requiring fixed surroundings (Figs. 2 and 3), the device was placed in indoor air with air conditions monitored continuously (thermometers and humidity meters, COS-03). In short-term measurements requiring different humidity (Fig. 4), we tested the device within a closed container and used ultrasonic humidifier or CaCl_2_ (AR, Sinopharm Chemical Reagent Co., Ltd) to adjust the humidity before carrying out the experiments.

## Supplementary information

Supplementary information

Description of Additional Supplementary Files

Supplementary Movie 1

Supplementary Movie 2

Supplementary Movie 3

Supplementary Movie 4

Supplementary Movie 5

Supplementary Movie 6

Supplementary Movie 7

## Data Availability

The authors declare that the main data supporting the findings of this study are contained within the paper and the Supplementary information files. All other relevant data are available from the corresponding author upon reasonable request.

## Code availability

The simulation files that support the plots and data analysis within this paper are available from the corresponding author upon request.
