# Supplementary material for: Circadian humidity fluctuation induced capillary flow for sustainable mobile energy
Source: Nat Commun. 2022 Mar 11;13:1291. doi: 10.1038/s41467-022-28998-y (PMC8917138; doi:10.1038/s41467-022-28998-y)
Supplement: Supplementary file 1 — Supplementary information [file 41467_2022_28998_MOESM1_ESM.pdf]

**Circadian humidity fluctuation induced capillary flow for sustainable mobile energy**

Jiayue Tang[1], Yuanyuan Zhao[2], Mi Wang[3,4], Dianyu Wang[5], Xuan Yang[5], Ruiran Hao[6], Mingzhan Wang[7], Yanlei Wang[3,4], Hongyan He[3,4]*, John H. Xin[2], Shuang Zheng[8]*

[1]Department of Chemistry, Hong Kong University of Science and Technology, Hong Kong, China

[2]Institute of Textiles & Clothing, Hong Kong Polytechnic University, Hong Kong, China

[3]Beijing Key Laboratory of Ionic Liquids Clean Process, Institute of Process Engineering, Chinese Academy of Sciences, Beijing 100190, China

[4]University of Chinese Academy of Sciences, Beijing 100049, China

[5]Beihang University, Beijing 100191, China

[6]School of environmental engineering, Yellow River Conservancy Technical Institute, Kaifeng 475004, China

[7]Pritzker School of Molecular Engineering, University of Chicago, Chicago, Illinois 60637, USA

[8]Department of Biomedical Sciences, City University of Hong Kong, Hong Kong, China

*Correspondence and requests for materials should be addressed to S. Z. (Email: zhengshuang@iccas.ac.cn) and H. H. (hyhe@ipe.ac.cn)

**Content**

**Supplementary Figure 1**. Fabrication and characterization of the nanowire array with electrode.

**Supplementary Figure 2**. Power generation of the drop generator.

**Supplementary Figure 3**. Complete flow induced by moisture absorption/desorption.

**Supplementary Figure 4**. Velocity fluctuation quantified using standard deviation ($SD$) and relative standard deviation ($RSD$) under different air $RH$.

**Supplementary Figure 5**. Air humidification caused flow accelerating and voltage improvement.

**Supplementary Figure 6**. Far-infrared spectra for $x(H_2O)$ is 0, 4, 13 respectively.

**Supplementary Figure 7**. Schematic illustration of the flow caused ion accumulation.

**Supplementary Figure 8**. Snapshots of water containing ILs confined between modified PDMS basements.

**Supplementary Figure 9**. Moisture-triggered $Omim^+$ clusters across PDMS nanowire array.

**Supplementary Figure 10**. Wind enhanced power generation.

**Supplementary Figure 11**. Wind enhanced moisture absorption/desorption.

**Supplementary Figure 12**. Humidity fluctuation as a practical power resource.

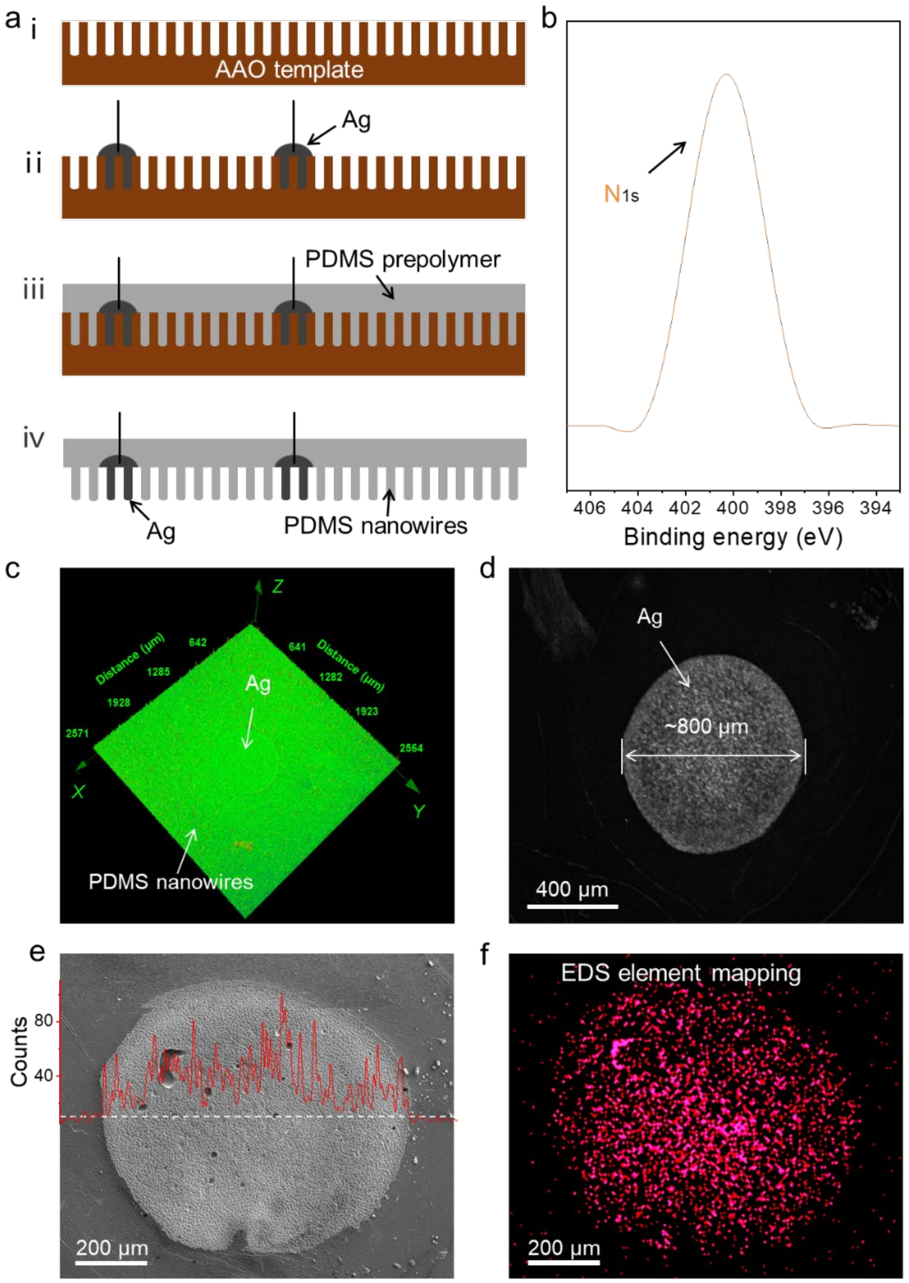


**Supplementary Fig. 1 Fabrication and characterization of the nanowire array with electrode. a**, Device preparation. Liquid silver-filled adhesive (ii) was added to AAO template (i). After heat curing of the conductive adhesive, PDMS prepolymer mixture was poured onto the AAO (iii), which was cured under 80 °C for 3 hours. Finally, the AAO template was removed (iv). **b**, X-ray photoelectron spectroscopy (XPS) analysis confirming successful modification of amine. **c,d,** Confocal laser scanning microscope imaged PDMS nanowire array and Ag/AgCl electrode with the diameter of ~800 μm. **e,**

Low-magnification SEM image of the electrode. The inset showed Ag content obtained by EDS linear scanning along the dotted white line over the electrode. **f**, EDS element mapping of **e** confirming Ag element.

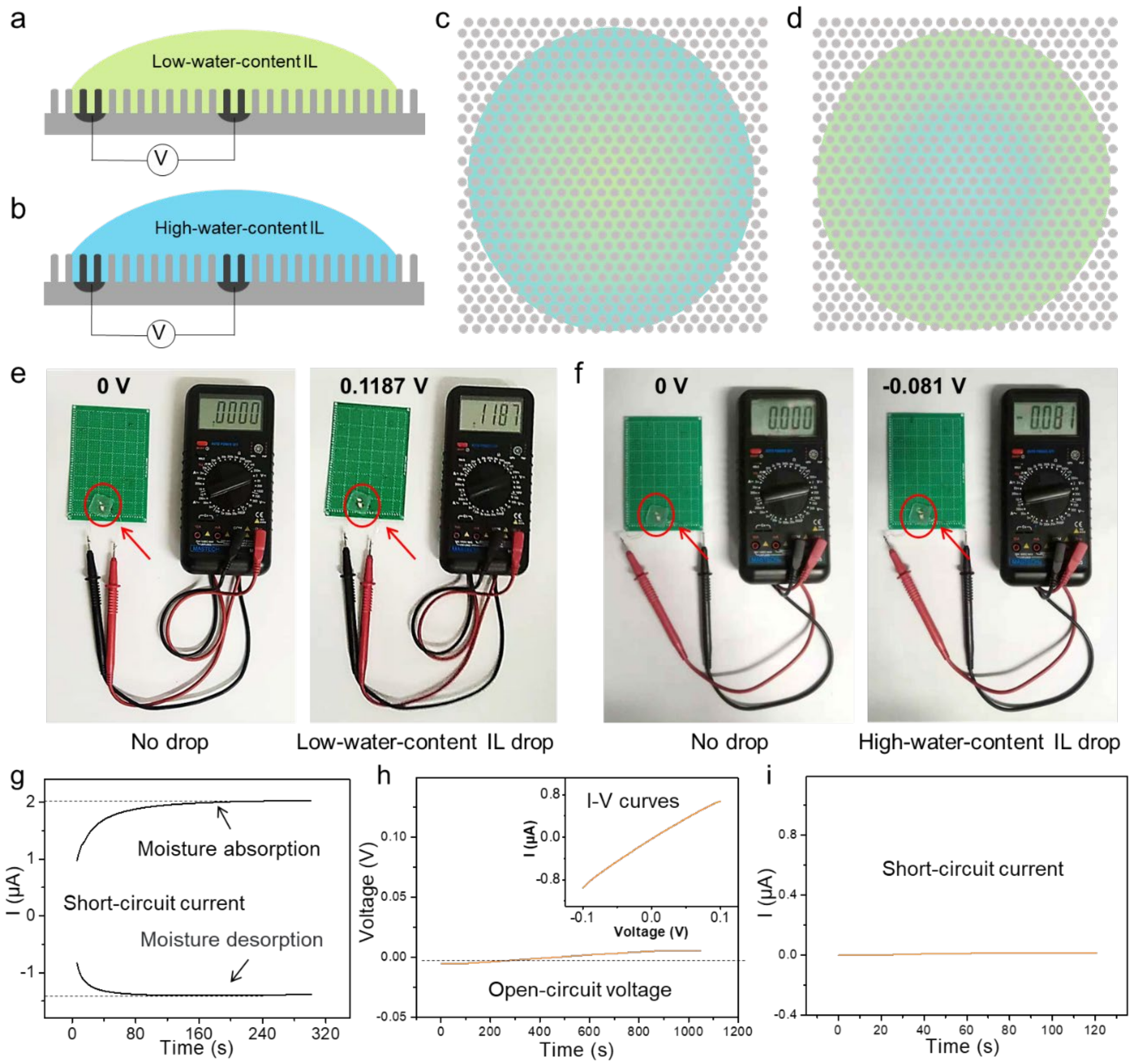


**Supplementary Fig. 2 Power generation of the drop generator. a,b**, Schematic illustration of dry and wet IL drop pinned on PDMS nanowire array. **c,d**, Top-view of **a** and **b** exposed to a common $RH$ of ~40% at 25 °C. **e,f**, A hand-held multimeter to further confirm output voltage of **a** and **b**. **g**, $I_{sc}$ of **a** and **b** measured with digital multimeter. **h,i**, $V_{OC}$, I-V curves and $I_{sc}$ for an IL drop with $WC$ equal to $WC_S$.

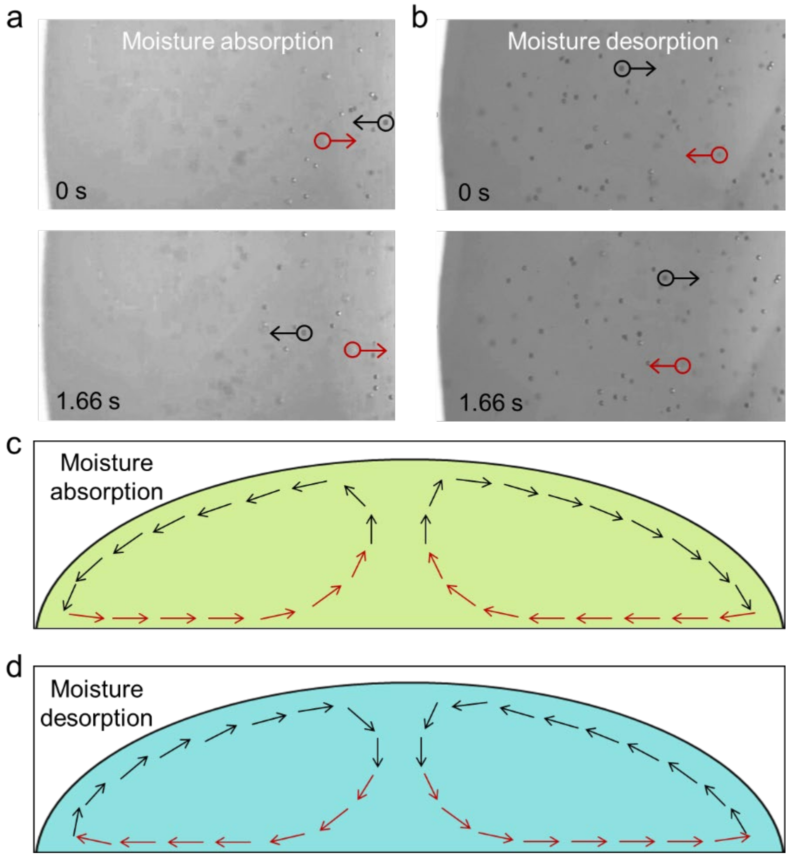


**Supplementary Fig. 3 Complete flow induced by moisture absorption/desorption. a,b,** Snapshots of the circulating flow. Red and black arrows represent microsphere movement direction at the lower and upper layer. **c,d,** Schemes of the circulating flow.

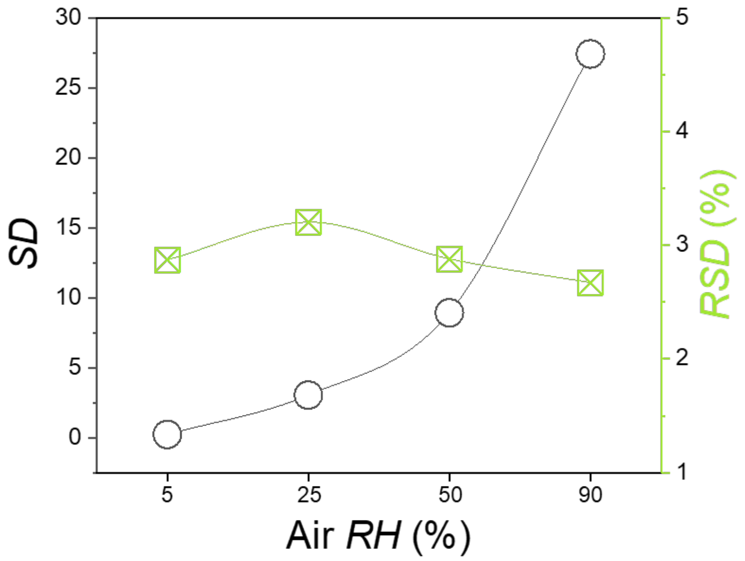


**Supplementary Fig. 4 Velocity fluctuation quantified using standard deviation (*SD*) and relative standard deviation (*RSD*) under different air *RH*.**

In statics, $RSD$ is the ratio of the standard deviation to the mean value, representing the extent of variability in relation to the mean of the population. The $RSD$ is useful because the $SD$ of data must always be understood in the context of the mean values. As such, for comparison between data with widely different mean values, $RSD$ is always used instead of $SD$.

From Supplementary Fig. 4, we find that the $RSD$ is almost the same within the four tests, showing the reliability of this velocity statistics. The $SD$ difference is attributed to the great differences of velocity mean values.

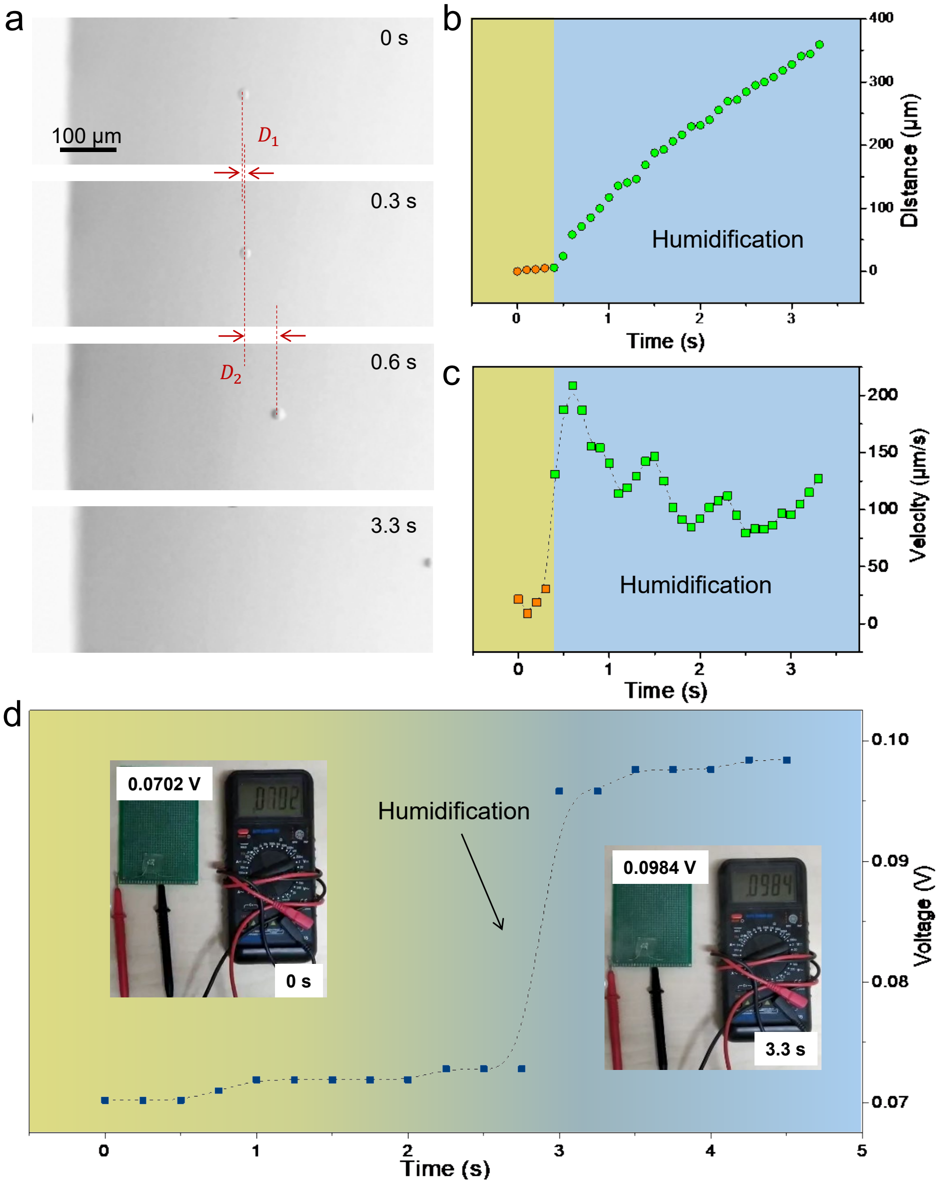


**Supplementary Fig. 5 Air humidification caused flow accelerating and voltage improvement. a,** Snapshots of the microsphere accelerated by dynamic improvement of environment humidity. **b,c,** Statistics of motion displacement and velocity versus time in **a**. **d,** Humidification induced rise in output voltage. Insets are snapshots of the hand-held multimeter showing $V_{OC}$.

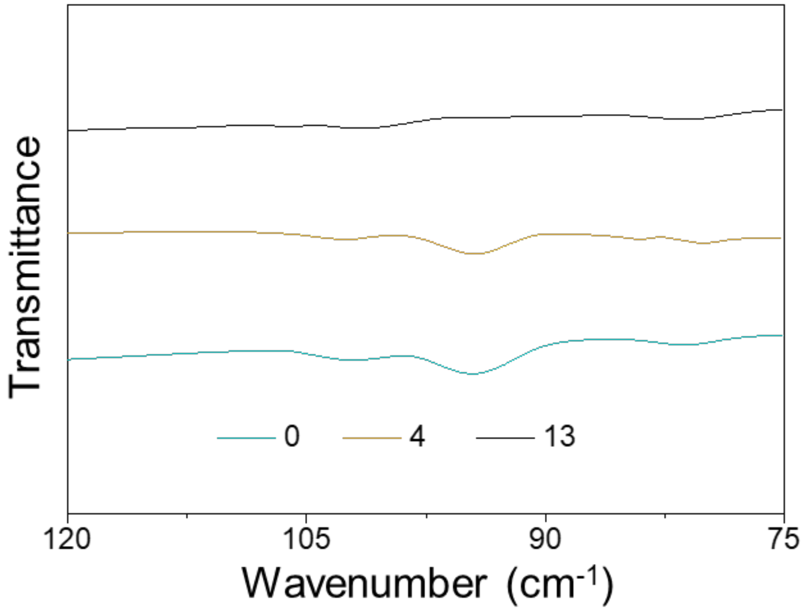


**Supplementary Fig. 6 Far-infrared spectra for $x(H_2O)$ is 0, 4, 13 respectively. a,** Weakening and disappearance of absorption peak at ~95 $cm^{-1}$ indicate moisture-loosed $Omim^+$ and $Cl^-$ ion pairs.

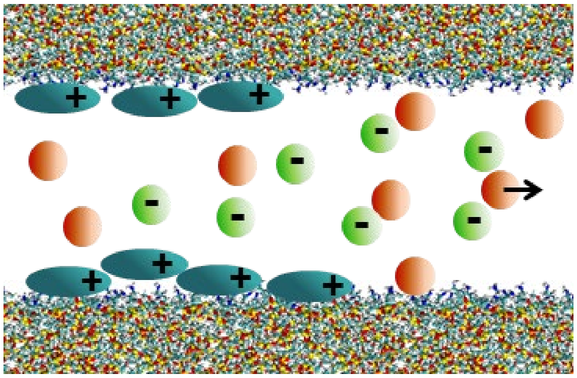

**Supplementary Fig. 7 Schematic illustration of the flow caused ion accumulation.** Blue ellipsoids, green balls and red balls represent Omim+, $Cl^-$ and $H_2O$, respectively.

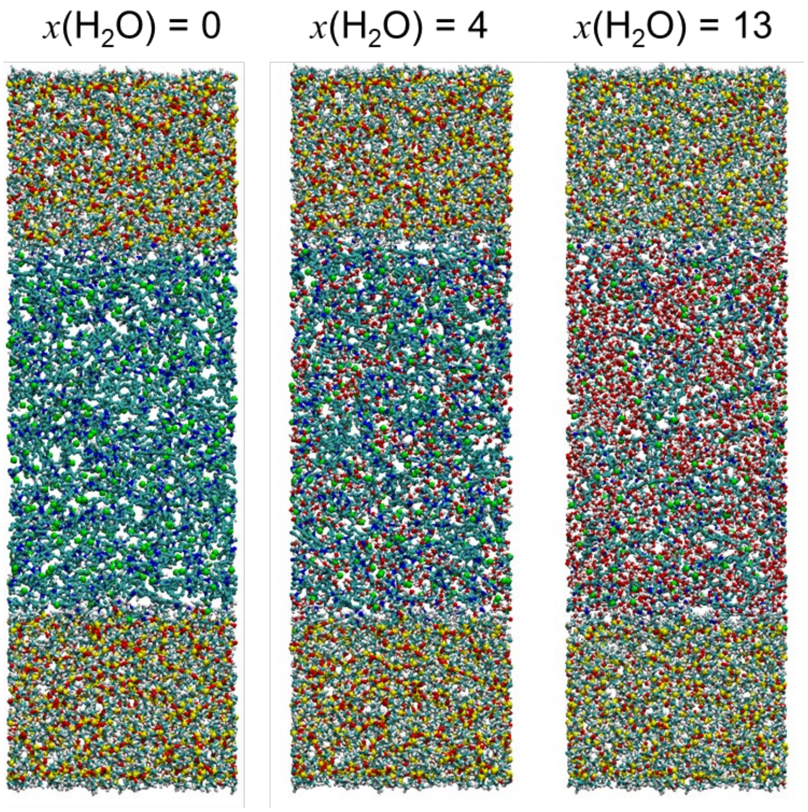


**Supplementary Fig. 8 Snapshots of water containing ILs confined between modified PDMS basements.** The blue, cyan, white, green, yellow, and red balls represent N, C, H, Cl, Si, and O atoms, respectively. From left to right, the content of water $x(H_2O)$ in the ILs is 0, 4 and 13.

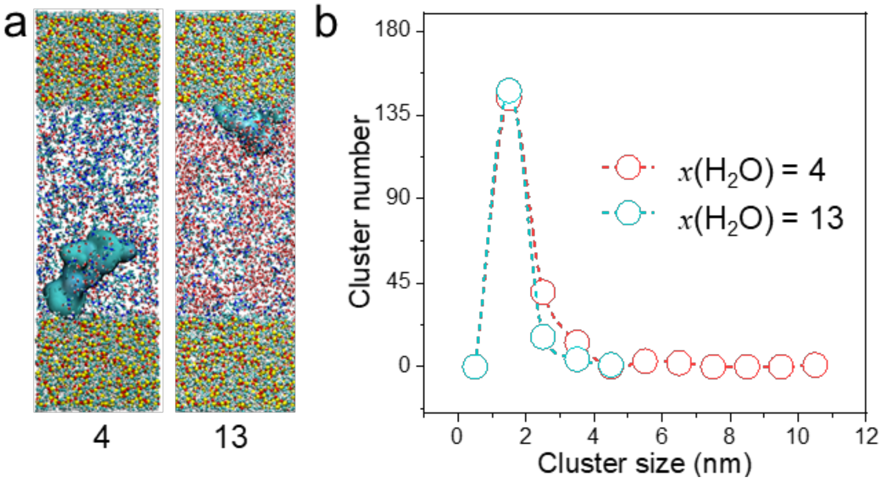

**Supplementary Fig. 9 Moisture-triggered Omim$^+$ clusters across PDMS nanowire array.** Snap structures and Omim$^+$ clusters size distribution when $x(H_2O)$ is 4 and 13 respectively.

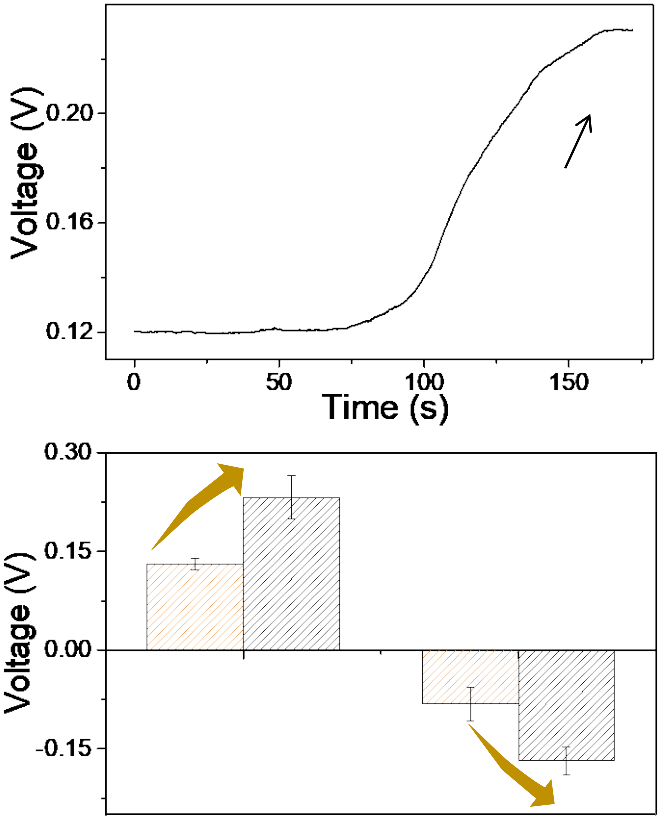


**Supplementary Fig. 10 Wind enhanced power generation. a**, $V_{OC}$ versus time recorded from dry drop exposed to wind (~2.5 km/h) with the $RH$ of ~40% at 25 °C. **b**, $V_{OC}$ of device in Fig. 2 could be further improved by wind (~2.5 km/h).

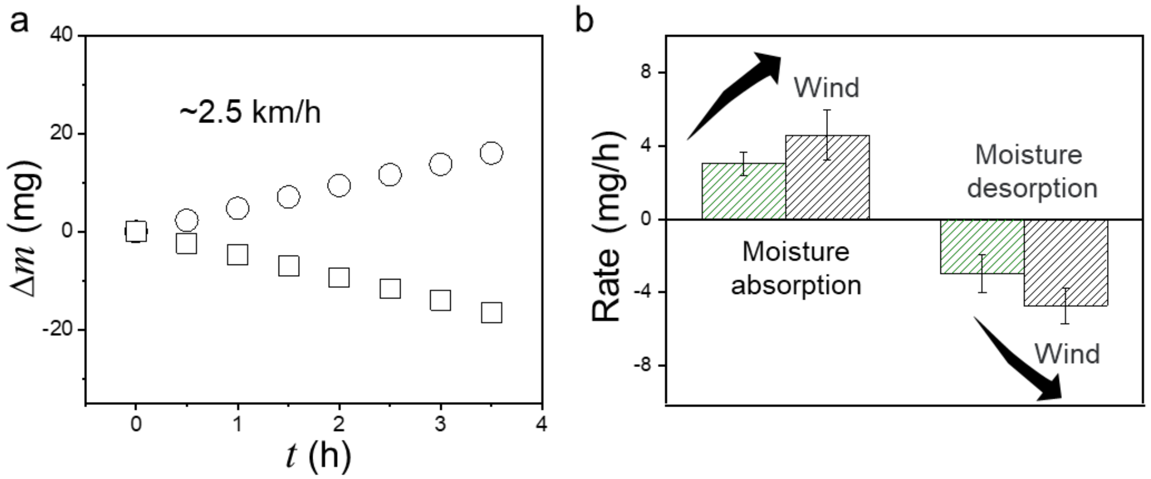


**Supplementary Fig. 11 Wind enhanced moisture absorption/desorption. a**, Moisture absorption/desorption of dry/wet IL drop exposed to air *RH* of ~40% with wind speed set to ~2.5 km/h. **b**, Calculated moisture absorption/desorption rate of both drops with and without wind.

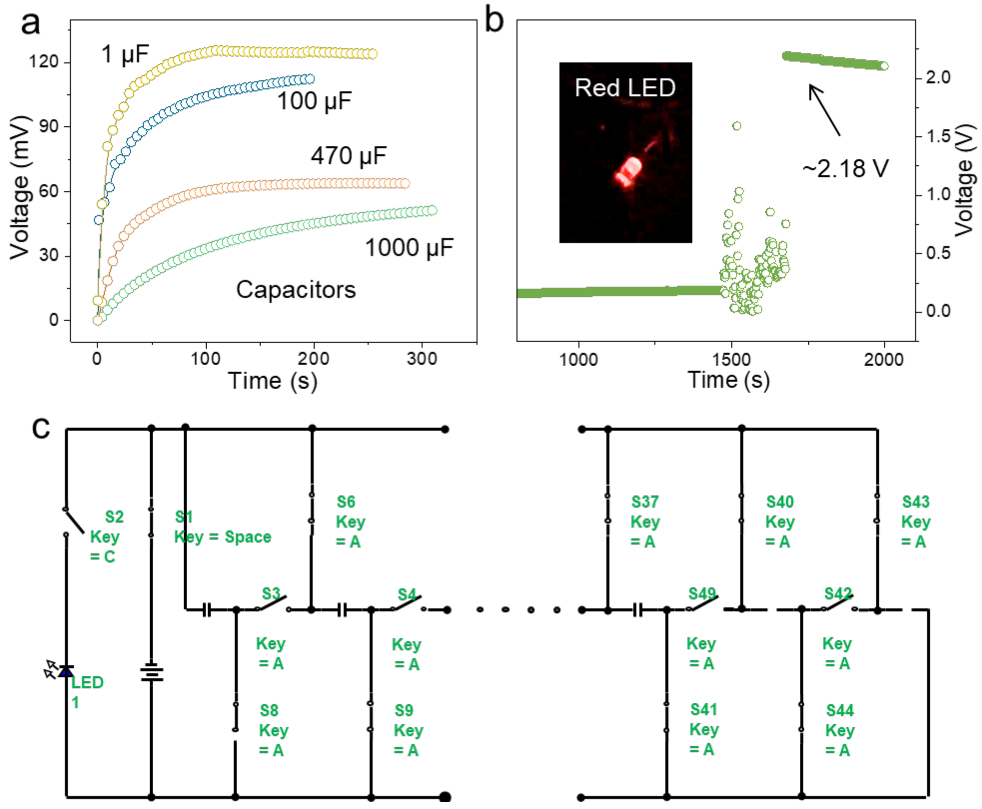


**Supplementary Fig. 12 Humidity fluctuation as a practical power resource. a**, Commercial capacitors charged by a single IL drop. **b,c**, A circuit for connecting 16 capacitors (22 μF) in series in order to output high voltage. Inset in **b** is a red LED powered by the drop-charged circuit in **c**.
