# Supplementary material for: Circadian humidity fluctuation induced capillary flow for sustainable mobile energy
Source: Nat Commun. 2022 Mar 11;13:1291. doi: 10.1038/s41467-022-28998-y (PMC8917138; doi:10.1038/s41467-022-28998-y)
Supplement: Supplementary file 2 — Description of Additional Supplementary Files [file 41467_2022_28998_MOESM2_ESM.pdf]

Description of Additional Supplementary Files

File name: Supplementary Movie 1.

Description: Polystyrene microspheres (diameter, ~18 μm) loaded dry IL drop (75 μL) deposited on chemically modified PDMS nanowire array with the diameter of ~90 nm and length of ~2 μm. The high-magnification microscope imaging has revealed the bottom inward flow from drop triple-phase contact line to the bottom center.

File name: Supplementary Movie 2.

Description: Polystyrene microspheres (diameter, ~18 μm) loaded wet IL drop deposited on chemically modified PDMS nanowire array with the diameter of ~90 nm and length of ~2 μm. The high-magnification microscope imaging has revealed the bottom outward flow from the bottom center to drop triple-phase contact line.

File name: Supplementary Movie 3.

Description: Low-magnification microscope imaging of polystyrene microspheres (diameter, ~18 μm) loaded dry IL drop (75 μL) deposited on chemically modified PDMS nanowire array (diameter of ~90 nm and length of ~2 μm) to observe the whole cyclic flows including the bottom inward flow in Supplementary Movie 1 and the inverse flow along the top layer of the drop caused by the Gibbs–Marangoni effect.

File name: Supplementary Movie 4.

Description: Low-magnification microscope imaging of polystyrene microspheres (diameter, ~18 μm) loaded wet IL drop deposited on chemically modified PDMS nanowire array (diameter of ~90 nm and length of ~2 μm) to observe the whole cyclic flows including the bottom outward flow in Supplementary Movie 2 and the inverse flow along the top layer of the drop caused by the Gibbs–Marangoni effect.

File name: Supplementary Movie 5.

Description: Optical imaging of humidification induced rise in output voltage confirmed humidity as the cause of directional flow.

File name: Supplementary Movie 6.

Description: Microscope imaging of IL drop carried polystyrene microspheres (diameter, ~18 μm) accelerated by dynamic improvement of environment humidity.

File name: Supplementary Movie 7.

Description: Connected and disconnected drop array with LCD screen on and off.
